# Postmenopausal breast cancer in Iran; risk factors and their population attributable fractions

**DOI:** 10.1186/1471-2407-12-414

**Published:** 2012-09-19

**Authors:** Reza Ghiasvand, Shahram Bahmanyar, Kazem Zendehdel, Sedigheh Tahmasebi, Abdolrasoul Talei, Hans-Olov Adami, Sven Cnattingius

**Affiliations:** 1 Cancer Research Center, Cancer Institute of Iran, Tehran University of Medical Sciences, Tehran, Iran; 2 Clinical Epidemiology Unit, Department of Medicine, Karolinska Institutet, Stockholm, Sweden; 3 Department of Medical Epidemiology and Biostatistics, Karolinska Institutet, Stockholm, Sweden; 4 Department of Surgery, Shahid Faghihi Hospital, Shiraz University of Medical Sciences, Shiraz, Iran; 5 Department of Epidemiology, Harvard School of Public Health, Boston, MA, USA

**Keywords:** Breast neoplasm, Postmenopausal, Middle East, Risk factor, Attributable fraction

## Abstract

**Background:**

Causes of the rapidly increasing incidence of breast cancer in Middle East and Asian countries are incompletely understood. We evaluated risk factors for postmenopausal breast cancer and estimated their attributable fraction in Iran.

**Methods:**

We performed a hospital-based case–control study, including 493 women, diagnosed with breast cancer at 50 years or later between 2005–2008, and 493 controls. We used logistic regression models to estimate multivariable odds ratios (OR) and 95% confidence intervals (CI), and population attributable fractions (PAF) for significant risk factors.

**Results:**

The risk of breast cancer decreased with increasing parity. Compared with nulliparous women, the adjusted OR (95% CI) was 0.53 (0.25-1.15) for parity 1–3, 0.47 (0.29-0.93) for parity 4–6 and 0.23 (0.11-0.50) for parity ≥7. The estimated PAF for parity (<7) was 52%. The positive association between body mass index (BMI) and breast cancer risk was confined to women diagnosed at 58 years or later. Compared with normal weight women (BMI 18.5-24.9), overweight (BMI 25–29.9) and obese (BMI ≥30) women were at increased risk of breast cancer diagnosed at 58 years or later (ORs [95% CI] 1.27 [0.97-2.65] and 2.34 [1.33-4.14], respectively). The estimated PAF for obesity/overweight (BMI >25) was approximately 25%. The family history was significantly associated with increased breast cancer risk, but not increasing height, early age at menarche, late age at first birth or short breastfeeding.

**Conclusions:**

Decreasing parity and increasing obesity are determinants of increasing breast cancer incidence among Iranian women. These trends predict a continuing upward trend of postmenopausal breast cancer.

## Background

With an estimated 1.4 to 1.6 million new cases diagnosed around the world in 2008, breast cancer is the most common cancer among women in high-and middle-income, but also in a growing number of low-income countries [1]. There is, however, still a considerable geographical variation in incidence [2,3], especially with respect to postmenopausal breast cancer [4]. Until recently, Middle East and Asian countries had among the lowest rates of breast cancer in the world [5], but the incidence has increased rapidly during the past two decades [4,6,7]. This increase is expected to continue and breast cancer mortality will almost double in the Middle East during the coming decade, which is the highest worldwide expected increase [5].

In Iran, the annual number of new breast cancer cases is estimated to double from 2008 to 2030 due to the demographic transition alone [8]. Current knowledge about breast cancer etiology is primarily derived from studies in high-income countries [9]. However, the wide variety of lifestyle and environmental exposures in low- and middle-income countries in transition might provide novel information on breast cancer etiology [10]. In particular, Middle East, a large geographic area with a variety of ethnic groups, is now undergoing a rapid transition in lifestyle and reproductive patterns. For example, the average number of childbirths in Iran declined from over 7 per woman in the early 1980s to 1.7 in 2007 [11,12]. Lack of relevant epidemiological data makes it difficult to understand the reasons for the lower incidence of breast cancer in the Middle East and Asia [13,14] compared with western countries, and to estimate and interpret the contribution of changing pattern of established risk factors in the recent rapidly increasing trend of breast cancer in these populations [10,14]. To this end, we undertook a large case–control study in Iran to evaluate some established risk factors of postmenopausal breast cancer and their role in the rising trend of breast cancer in Iran.

## Methods

We performed a hospital-based case control study to identify risk factors for postmenopausal breast cancer in Iranian women. Cases were recruited from Motahari Breast Clinic of Shiraz University of Medical Sciences. This center collects data from about 80% of all incident breast cancer patients treated in main hospitals of Shiraz city, including general hospitals of Faghihi and Namazi and private hospitals of Dena and Ordibehesht. Shiraz, the capital city of Fars province, is located in the southern part of Iran. Faghihi hospital is the major hospital providing oncology services for breast cancer patients, and more than 85% of newly diagnosed breast cancer patients within the Fars province are referred to this hospital for treatment. In addition, the Faghihi hospital is also the referral center for other provinces in the southern part of Iran, including Bushehr, Khuzestan, Hormozgan and Kohgiluye & Boyer Ahmad. Women with histopathologically confirmed breast cancer are referred to Motahari clinic for postoperative care and follow-ups. Women with newly diagnosed breast cancer are interviewed using a structured questionnaire that contains questions about risk factors for breast cancer and other reproductive cancers.

Eligible cases were women with an incident histopathologically confirmed breast cancer, diagnosed at 50 years of age or older. Most patients (93%) were interviewed within six months after diagnosis. All women were interviewed between September 2005 and December 2008. We excluded five cases with missing information on age and eleven case patients from other provinces because we could not find any matched controls for them.

Controls were frequency-matched with cases on five-year age groups and province of residence. Controls were primarily selected from healthy female visitors accompanying patients referred to the Faghihi hospital for general surgery (60%), urology (24%) and cardiovascular (16%) diseases. Controls did not have a history of breast cancer at enrollment. For a small number of old case patients (11%), for whom we could not find a healthy control, we selected controls from patients referred to urology, cardiovascular and general surgery wards for conditions unrelated to known or likely risk factors for breast cancer, like gynecologic, hormonal or neoplastic diseases. A total of 487 potentially healthy visitor controls and 98 patient controls were selected, but 49 (10%) healthy women and 43 (44%) patients refused to participate. Finally, a total of 493 cases of postmenopausal breast cancer and 493 controls were included in the study.

Reliable information about age at menopause was not available for the study subjects. When information on menstrual history is not available, 50 years of age may be the best proxy for all menses-based definitions of menopause in western countries[15]. It has been shown that the mean age of natural menopause among Iranian women is approximately 48 years [16,17]. Therefore, we used 50 years as a conservative cut point for defining postmenopausal breast cancer.

### Data collection

For both cases and controls, face-to-face interviews were performed, using an identical structured questionnaire to collect information on age, place of residence, marital status, educational level, family history of breast cancer, age at menarche, occupation, parity, past use of oral contraceptives, age at first pregnancy and lifetime duration of breastfeeding. Cases were interviewed at their first time of treatment between September 2005 and December 2008. Controls were interviewed from May 2009 through August 2009. Interviews were conducted by two trained female nurses (one for cases and one for controls), and the time of interviews was similar for cases and controls. Height and weight were measured at the end of the interview. None of interviewers were aware of the study hypotheses.

All included subjects were informed about the study protocol and provided oral informed consent. The study was approved by the Research Ethics Committee of Shiraz University of Medical Sciences.

### Exposure categorization

Information on parity was collected as a categorical variable (0, 1–3, 4–6, ≥7), and therefore analyzed accordingly. Information on age at menarche and age at first childbirth was collected using categorical variables in the questionnaire: < 12, 12–15 and >15 years for age at menarche; and <15, 15–24 and ≥25 years for age at first childbirth. Education was categorized as illiterate, elementary, high school and university degree. Occupation was grouped as housewife and employed. Life time duration of breastfeeding was categorized as never and in approximate tertiles based on the distribution among controls (1–94, 95–162, and more than 162 months). Average months of breastfeeding per child was categorized as <12 months and ≥12 months. Oral contraceptive use was categorized as never/ever, and (among ever users) the duration of usage, categorized based on the tertile values of the distribution among controls (1–48, 49–116, and more than 116 months). Height was categorized based on its approximate quartile cut-off points of the distribution among controls (≤151, 152–156, 157–160 and >160 cm). BMI was calculated as weight (kg) divided by height squared (m^2^) and categorized as <18.5, 18.5-24.9, 25–29.9 and ≥30 kg/m^2^.

### Statistical analyses

We used unconditional logistic regression with adjustment for the matching factors (age and residential place) to estimate the odds ratios (ORs) and 95% confidence intervals (CI). We used univariable analyses to estimate the crude ORs and 95% CI. Multivariable logistic regression models were fitted to estimate the association between each independent variable and postmenopausal breast cancer while adjusting for other potential confounding variables. Additional analyses were performed on data restricted to parous women (n = 936). The models for these analyses were stratified for categories of parity.

To examine the hypothesis that the obesity-related risk is smaller among perimenopausal or recently postmenopausal women than among elderly women, we also conducted analyses stratified by the mean of age of breast cancer (less than 58 years and 58 years or older). We assessed significance of interaction using Wald test statistics. We also computed adjusted population attributable fractions and 95% confidence intervals for significant variables (parity, BMI, family history of breast cancer and oral contraceptive use) as described by Greenland and Drescher [9], using *aflogit* module developed for Stata. All P values were 2-sided. All analyses were conducted using Stata 11.0 software (Stata Corporation, College Station, TX).

## Results

The mean age of the 493 women diagnosed with postmenopausal breast cancer was 58.2 (±7.2 standard deviation [SD], median = 56, range: 50 – 89) and 58.0 (±7.4 SD, median = 56, range: 50 – 88) among the 493 frequency-matched controls. As depicted in Table 1, illiteracy rates were 70% among cases and 79% among controls with parity 7 and higher. Corresponding rates among women with parity 1 to 3 were 19% among cases and 30% among controls. While 45% of cases and 29% of controls with parity 1–3 were employed, only 1% of both cases and controls with parity 7 or higher were employed. Moreover, obesity (BMI ≥30) was generally more prevalent among those with lower parity. Table 2 shows that the distribution of parity and literacy rates among the controls mirror those reported from general female population in the 2006 census in the Fars Province, and are also similar to overall rates in Iran [18].

**Table 1:** Distribution of cases and controls participated in the study in Southern Iran according to selected variables by parity

	**Parity**							
	**Nulliparous**		**1-3**		**4-6**		**7+**	
	**Cases**	**Controls**	**Cases**	**Controls**	**Cases**	**Controls**	**Cases**	**Controls**
	**(n = 38)**	**(n = 12)**	**(n = 130)**	**(n = 76)**	**(n = 227)**	**(n = 197)**	**(n = 98)**	**(n = 208)**
	**n (%)**	**n (%)**	**n (%)**	**n (%)**	**n (%)**	**n (%)**	**n (%)**	**n (%)**
**Age at menarche (years)**								
< 12	7 (18)	0 (0)	15 (12)	6 (8)	17 (7)	5 (2)	3 (3)	14 (7)
12 – 15	26 (69)	11 (92)	98 (75)	66 (87)	188 (83)	169 (86)	83 (85)	179 (86)
>15	5 (13)	1 (8)	17 (13)	4 (5)	22 (10)	23 (12)	12 (12)	15 (7)
**Education**								
None	15 (39)	6 (50)	24 (19)	23 (30)	71 (31)	99 (50)	69 (70)	164 (79)
Elementary	6 (16)	2 (17)	21 (16)	12 (16)	102 (45)	69 (35)	26 (27)	40 (19)
High school	5 (13)	4 (33)	56 (43)	27 (36)	44 (20)	27 (14)	3 (3)	4 (2)
University	12 (32)	0 (0)	29 (22)	14 (18)	10 (4)	2 (1)	0 (0)	0 (0)
**Occupation**								
Housewife	20 (53)	10 (83)	72 (55)	54 (71)	202 (89)	192 (97)	97(99)	206 (99)
Employed	18 (47)	2 (17)	58 (45)	22 (29)	25 (11)	5 (3)	1 (1)	2 (1)
**Height (cm)**								
<152	11 (29)	8 (67)	40 (31)	16 (21)	56 (25)	49 (25)	36 (37)	59 (28)
152 – 156	14 (37)	2 (17)	36 (28)	18 (24)	85 (37)	49 (25)	25 (26)	53 (26)
157 – 160	7 (18)	1 (8)	25 (19)	25 (33)	43 (19)	67 (34)	15 (15)	70 (34)
>160	4 (11)	1 (8)	24 (18)	17 (22)	39 (17)	32 (16)	18 (18)	26 (12)
Missing	2 (5)	0 (0)	5 (4)	0 (0)	4 (2)	0 (0)	4 (4)	0 (0)
**BMI**								
<18.5	0 (0)	0 (0)	0 (0)	2 (3)	1 (0)	5 (3)	3 (3)	7 (3)
18.5 – 24.9	11 (29)	5 (42)	37 (28)	22 (29)	50 (22)	69 (35)	27 (28)	77 (34)
25 – 29.9	19 (50)	7 (58)	52 (40)	30 (39)	97 (43)	77 (39)	40 (41)	79 (38)
≥30	6 (16)	0 (0)	36 (28)	22 (29)	75 (33)	46 (23)	24 (24)	51 (25)
Missing	2 (5)	0 (0)	5 (4)	0 (0)	4 (2)	0 (0)	4 (4)	0 (0)

**Table 2:** Distribution of selected population indicators in Iran, Fars Province and control group in the present study

**Indicator**	**Iran**^**1**^	**Fars Province**^**1**^	**Present Study**
**Average number of childbirths per woman**			
50 – 54 years	5.42	5.63	5.61
55 – 59 years	5.93	6.06	6.02
60 – 64 years	6.18	6.16	6.27
65+	6.11	6.03	6.57
**Female literacy rate**			
Total (6 years and older)	84.6	86.6	-
50 – 64 years	41.2	44.8	44.9
65+	16.1	16.9	21.2
**Urbanization**	68.4	61.1	59.6

^1^According to the 2006 census [16].

Compared with nulliparous women, parous women had a reduced risk of postmenopausal breast cancer in both univariable and multivariable analyses (Table 3). The risk of breast cancer decreased with increasing parity. Compared with nulliparous women, women with 1–3 and 4–6 childbirths had a 47 and 53% reduction in risk, while risk was reduced by 77% in women with at least 7 childbirths (OR = 0.23). Risk of postmenopausal breast cancer increased significantly with educational level in the univariable, but not in the multivariable analyses. This change in risk estimate was primarily due to confounding by parity; when we only adjusted for parity, we obtained similar risk estimates for education as those presented in the adjusted analysis in Table 3 (data not shown).

**Table 3:** Crude and adjusted odds ratios (OR) and 95% confidence intervals (CI) of the 493 cases and 493 controls in the study in Southern Iran

**Parameter**	**Events**	**Crude OR (95% CI)**	**Adjusted OR (95%CI)**^**1**^
	**Cases/Controls**		
**Parity**			
Nulliparous	38/12	1	1
1 – 3	130/76	0.50 (0.25 – 1.02)	0.53 (0.25 – 1.15)
4 – 6	227/197	0.34 (0.17 – 0.67)	0.47 (0.29 – 0.93)
7+	98/208	0.14 (0.07 – 0.28)	0.23 (0.11 – 0.50)
P for trend		< 0.001	< 0.001
**Age at menarche (years)**			
< 12	42/25	1.80(1.07 – 3.02)	1.72 (0.96 – 3.07)
12 – 15	395/425	1	1
> 15	56/43	1.40 (0.91 – 2.13)	1.11 (0.86 – 2.24)
P for trend		0.75	0.09
**Education**			
None	179/292	1	1
Elementary	155/123	2.05 (1.51 – 2.79)	1.33 (0.83 – 2.29)
High school	108/62	2.84 (1.95 – 4.12)	1.26 (0.78 – 2.03)
University	51/16	5.19 (2.81 – 9.59)	1.50 (0.64 – 3.48)
P for trend		< 0.001	0.06
**Occupation**			
Housewife	391/462	1	1
Employed	102/31	3.88 (2.51 – 5.99)	2.05 (1.15 – 3.92)
**Height (cm)**^**2**^			
≤ 151	143/132	1	1
152 – 156	160/122	1.21(0.86 – 1.69)	1.12 (0.77 – 1.62)
157 – 160	90/163	0.50(0.35 – 0.72)	0.73 (0.49 – 1.71)
> 160	85/76	1.03(0.69 – 1.52)	1.08 (0.69 – 1.66)
Missing	15/0		
P for trend		0.06	0.10
**BMI (Overall)**			
< 18.5	4/14	0.38 (0.12 – 1.19)	0.60 (0.17 – 2.11)
18.5 – 24.9	129/181	1	1
25 – 29.9	208/193	1.43 (1.06 – 1.95)	1.39 (1.02 – 1.94)
≥ 30	141/119	1.58 (1.12 – 2.22)	1.61 (1.18 – 2.30)
Missing	15/0		
P for trend		<0.001	0.01
**BMI (age <58 years)**			
< 18.5	4/5	0.90 (0.23 – 3.55)	-
18.5 – 24.9	78/90	1	1
25 – 29.9	126/123	1.18 (0.79 – 1.74)	1.27 (0.80 – 2.02)
≥ 30	89/82	1.25(0.81 – 1.91)	1.21 (0.73 – 1.99)
P for trend		0.30	0.47
**BMI (age ≥58 years)**			
< 18.5	0/9	-	-
18.5 – 24.9	47/77	1	1
25 – 29.9	82/70	1.91 (1.18 – 3.11)	1.56 (0.97 – 2.65)
≥ 30	52/37	2.30 (1.32 – 4.01)	2.34 (1.33 – 4.14)
P for trend		0.002	0.008
P for interaction^3^		0.16	0.09
**F.H. of breast cancer**			
No	422/470	1	1
Yes	71/23	3.50 (2.37 – 5.17)	2.61 (1.72 – 3.96)
1^st^ relative	43/15	3.19(1.73 – 5.86)	2.13 (1.20 – 4.46)
2^nd^ relative	28/8	3.89(1.74 – 8.69)	1.98 (0.96 – 5.62)
P for trend		< 0.001	0.003

(Abbreviation F.H.: Family History).

^1^Adjusted for all variables in the table in addition to age.

^2^Height was categorized based on approximate quartile values of the control population.

^3^Multiplicative interaction between BMI and age.

BMI revealed a significant linear association with breast cancer risk (P-trend = 0.01). Compared with normal weight women (BMI 18.5-24.9), overweight (BMI 25.0-29.9), and obese women (BMI ≥30) had, in the unadjusted and adjusted analyses, approximately a 40% and a 60% increased risk of breast cancer, respectively. In the association between BMI and breast cancer, parity was a weak positive confounder and only adjusting for parity slightly attenuated the OR related to overweight (OR = 1.39, 95% CI 1.02 – 1.94) but not obesity (OR = 1.61, 95% CI 1.18 – 2.30). In analyses stratified by the mean age of breast cancer (less than 58 years and 58 years or more), the positive association between BMI and breast cancer was restricted to women aged 58 or older. However, the (multiplicative) interaction term between BMI and age was not significant (Table 3). Family history of breast cancer was associated with increased risks of postmenopausal breast cancer. There was no linear association between age at menarche and risk of postmenopausal breast cancer (P for trend 0.09). Mean height for cases and controls were 154.8 cm (SD = 6.2) and 155.8 cm (SD = 6.3), respectively, but height did not influence risk of breast cancer in the adjusted analysis.

To further investigate the parity-related reduced breast cancer risk, we investigated effects of reproductive factors in parous women (Table 4). Compared to women with 1–3 births, risk of breast cancer was reduced among women with 4–6 births (OR = 0.74 CI: 0.50 – 1.09) and 7 or more births (OR = 0.30 CI: 0.20 – 0.47). Cases were more likely to have their first childbirth at 25 years or later, but in multivariable analysis we found no significant association between age at first childbirth and risk of postmenopausal breast cancer. In the univariable analysis, lifetime duration of breastfeeding showed a negative linear association with risk of breast cancer and women who breastfed for a longer time were at a decreased risk of postmenopausal breast cancer (P for trend < 0.001). However, in multivariable analysis, breastfeeding was no longer associated with risk of breast cancer. We also examined the average months of breastfeeding per child, which was not associated with breast cancer risk (Table 4). Compared with women never used oral contraceptives, women who ever used were at a higher risk of breast cancer. The association between oral contraceptive use and breast cancer risk was strengthened in the adjusted analysis, but there was no dose response relationship between length of oral contraceptive use and breast cancer risk.

**Table 4:** Crude and adjusted odds ratios (OR) and 95% confidence intervals (95% CI) for postmenopausal breast cancer risk factors among parous women

**Variable**	**Events**	**Crude OR**	**Adjusted OR**^**1**^
	**Case/control**	**(95%CI)**	**(95%CI)**
**Parity**			
Overall	455/481		
1 – 3	130/76	1	1
4 – 6	227/197	0.67 (0.47 – 0.94)	0.74 (0.50 – 1.09)
7+	98/208	0.27 (0.19 – 0.39)	0.30 (0.20 – 0.47)
P for trend		< 0.001	< 0.001
**Age at first childbirth**			
Overall	454/481		
<15	41/56	0.87 (0.56 – 1.34)	0.55 (0.28 – 1.10)
15 - 24	318/381	1	1
≥ 25	95/44	2.57 (1.75 – 3.79)	1.24 (0.40 – 3.80)
P for trend		< 0.001	0.77
**Breastfeeding (months)**			
Overall	454/481		
Never	21/20	0.63 (0.33 – 1.20)	0.93 (0.29 – 1.86)
1 – 94	268/162	1	1
95 – 162	131/149	0.53 (0.39 – 0.72)	0.87 (0.27 – 2.78)
> 162	34/150	0.13 (0.08 – 0.21)	0.32 (0.08 – 1.22)
P for trend		< 0.001	0.30
**Breastfeeding per child**			
Overall	454/479		
Never	21/20	0.91 (0.47 – 1.78)	0.98 (0.71 – 1.35)
< 12 months	125/110	1	1
≥ 12 months	308/349	0.77 (0.57 – 1.04)	0.92 (0.45 – 1.88)
P for trend		0.20	0.68
**OCP usage (months)**			
Overall	454/481		
Never	178/236	1	1
1 – 48	135/90	1.50 (1.15 – 1.95)	1.99 (1.03 – 3.83)
49 – 116	73/73	1.98 (1.42 – 2.77)	2.25 (1.12 – 4.49)
> 116	68/82	1.32 (0.90 – 1.93)	1.40 (0.66 – 2.99)
P for trend		0.36	0.10

^1^Adjusted for all variables in the table in addition to age, age at menarche, education, BMI and family history of breast cancer.

Population attributable fractions for statistically significant risk factors in the multivariable logistic models and combination of them are shown in Table 5. Parity less than 7 children and overweight/obesity (BMI > 25) accounted for about 53 and 25% of breast cancer among postmenopausal women, respectively. The estimated attributable fraction combined for all significant risk factors including parity (< 7), BMI (> 25), family history of breast cancer and oral contraceptive usage was 71%.

**Table 5:** **Percent population attributable fraction (PAF)**^**1**^**and 95% confidence intervals (CI) according to significant variables and their combination**

	**PAF (95% CI)**^**2**^
**Parity (< 7)**	52.6 (41.2 – 61.8)
**BMI (> 25)**	24.8 (8.3 – 38.3)
**Parity + BMI**	64.2 (52.1 – 73.2)
**Family history of breast cancer (FH)**	15.7 (10.6 – 20.5)
**Oral contraceptive usage (OC)**	13.7 (0.3 – 25.3)
**Parity + BMI + FH + OC**	71.3 (63.1 – 80.7)

^1^ The PAFs denote the proportion of an outcome in the population that would be prevented if the exposure were completely removed assuming the association was causal and all confounding accounted for.

^2^ Derived from multivariable logistic regression model.

## Discussion

In this hospital-based case–control study of Iranian women, multiparity was a strong protective factor for postmenopausal breast cancer. Family history of breast cancer, oral contraceptive use and increasing BMI were associated with increased risks of postmenopausal breast cancer, while there were no significant associations with age at menarche, age at first childbearing, height, education and breastfeeding. The estimated attributable fraction combined for parity less than 7 children and overweight/obesity (BMI > 25) accounted for approximately 64% of breast cancer among postmenopausal women in Iran.

To the best of our knowledge, this is the largest case–control study of postmenopausal breast cancer in Iran. Nonetheless, our study has some important limitations. Hospital-based case–control studies are particularly susceptible to selection bias [19]. We are confident that our case-women were representative for all incident breast cancers in our study area. However, because population-based sampling of controls is not feasible in Iran, we had to rely on less optimal approaches for control selection. Although the indicators shown in Table 2 are reassuring, there is no mechanism whereby we can document that our controls were indeed representative for the prevalence of risk factors in the person-time that gave rise to our cases. Differential misclassification of exposure is another possible limitation. Such misclassification could arise both because cases and controls were interviewed by different nurses and because they recall or report differently. Reassuringly, however, our main findings were related to measured height and weight and to number of children, arguably factors with little opportunity for misclassification. Hence, we feel confident that the strong associations we found in relation to BMI and parity cannot possibly have arisen due to differential misclassification. In addition, we did not collect information about hormone replacement therapy (HRT), an established risk factor for breast cancer [9]. However, similar to many Asian countries [20,21], less than one per cent of Iranian women aged 50 years and older used HRT, often following surgical menopause [22]. Thus, HRT is an unlikely confounder in our study. Previous studies focusing on the association between parity and breast cancer have primarily been conducted in high-risk western countries among women with generally low parity and therefore limited possibilities to explore the association between parity and breast cancer in detail. A population-based study in Finland found that women with at least five births had a significantly decreased risk of breast cancer (SIR = 0.55), especially postmenopausal breast cancer [23], and a similar reduction in risk was reported for women with at least seven childbirths in Nigeria [24]. In our study, the protective effect of women with at least 7 childbirths was even stronger than the risk reductions reported from Finland and Nigeria. Socio-cultural differences may partly account for the differences of the parity-related reduced breast cancer risk between study populations.

We found that the grand multiparous women differed from those with fewer childbirths; they were less educated, had a younger age at first pregnancy, breastfed their children for longer periods and were less likely to use oral contraceptives. We find it likely that the protective effect of grand multiparity can to a large extent be explained by unmeasured lifestyle factors, which distort the association away from the null and strengthened it. For example, differences between women with high and low parity with respect to exposures to traditional low calorie high fiber diets, physical activity due to traditional way of life, early life events and conditions may play a role for the breast cancer risk in parous women [25]. Further knowledge about such differences in Iranian women with high and low parity may provide important clues with respect to prevention of late onset breast cancer.

In most studies, the association between parity and breast cancer risk is modified by age at first pregnancy. MacMahon et al. discovered that younger age at first childbearing reduces the risk of breast cancer [26]. This association is biologically plausible and has also been demonstrated in experimental rodent models [27]. The protective effect of age at first full-term pregnancy might be stronger for premenopausal than postmenopausal breast cancer [28]. Some epidemiological studies have, however, failed to reveal any relationship between breast cancer and age at first full-term pregnancy independent of parity [24,29]. We found no independent association between age at first childbirth and breast cancer. In the present study age at first pregnancy was in a lower range than in most other studies. For example, 90 percent of our controls had their first pregnancy before 25 years of age compared with 35 and 68 percent respectively in studies in the US [26] and New Zealand [29].

We found no significant association between breastfeeding and risk of postmenopausal breast cancer. Results of a collaborative study, combining epidemiological data from 47 studies, found a protective role of breastfeeding on the risk of breast cancer [30]. However, a meta-analysis [31] and several studies [32-34] have shown that the weak protective effect of lactation is confined to premenopausal breast cancer. In our previous study of premenopausal breast cancer in Iranian women, we found a protective effect of breast feeding [35].

We found no significant association between education and breast cancer after adjusting for other variables in multivariable analysis. In Iran, womens’ literacy rates increased from 57% to 97% between 1966 and 1996 in urban areas, while corresponding increase in rural areas was even more dramatic (from 5% to 86%) [36]. In 1998, 52% of students admitted to the governmental universities were women, which increased to 65% in 2007 [36]. Although education may have not directly affected breast cancer in Iran, the dramatic increasing rate of educated women is related to postponed marriage and childbearing, more oral contraceptive use and lower parity [12,37].

The positive association between BMI and risk of postmenopausal breast cancer in our study is consistent with the results of other studies in high-risk and low-risk countries[38-40]. In stratified analysis, we also found that this association was restricted to older women, while there was no significant association between BMI and breast cancer diagnosed below 58 years. A recent national survey revealed that 67% of Iranian women in the age group 55 to 64 years are overweight or obese (BMI ≥ 25) [41]. Among women aged 55 to 64 years, the prevalence of overweight and obesity was 64% among controls and 75% among cases (data not shown). In the report of International Obesity Task Force, Middle East has one of the highest prevalence rates of obesity in the world [42]. Hence, along with aging of the population, obesity will play a growing role in the burden of postmenopausal breast cancer in Iran and other Middle Eastern countries the coming decades. The present study showed that about 25% of postmenopausal breast cancers in Iran could be prevented if all women had BMI ≤ 25.

Iran experienced a high fertility rate until the 1980s [36]. In 1980, the total fertility rate was about seven children per women, which decreased to 2.8 children per woman in 1996 and 1.7 in 2007 [36,37]. It is estimated that, with assuming consistency of age-specific rates, Iran will, only due to the demographic transition, face a doubling in the number of new cases of breast cancer in 2030 [8]. However, this study indicates that 64% of postmenopausal breast cancer in Iran could be attributed to parity lower than 7 births and overweight/obesity (BMI > 25). Thus, with current decline in parity, increasing prevalence of obesity and social changes toward westernization, it is clear that due to increasing age-specific incidence we may expect an even more rapid growth, especially in postmenopausal women. Such trends may also be expected in other Middle Eastern and Asian countries with similar pattern of socio-cultural and demographic transitions. In addition to demographic differences, the declining parity in younger birth cohorts may also explain the current ten years lower mean age at onset of breast cancer in low- and middle-income countries compared with high-income countries [13,43-45].

## Conclusions

In summary, low parity and obesity were the main risk factors among postmenopausal women. The estimated attributable fraction combined for parity less than 7 children and overweight/obesity (BMI > 25) accounted for approximately 64% of breast cancer among postmenopausal women in Iran. We predict a birth cohort effect with an uprising trend in incidence rate of breast cancer in Iran and many other Middle Eastern countries in the near future.

## Abbreviations

BMI: Body mass index; CI: Confidence interval; OR: Odds ratio; SD: Standard deviation; PAF: Population attributable fraction.

## Competing interests

The author(s) declare that they have no competing interests.

## Authors’ contributions

RGH participated in the design of the study, performed the statistical analysis and drafted the manuscript. SHB contributed in the conception of the study and manuscript preparation. KZ contributed in the conception of the study and manuscript preparation. ST and AT contributed in the data acquisition and manuscript preparation. HOA contributed in the development of methodology and manuscript preparation. SC participated in the conception of the study, development of methodology and manuscript preparation. All authors read and approved the final manuscript.

## Pre-publication history

The pre-publication history for this paper can be accessed here:

http://www.biomedcentral.com/1471-2407/12/414/prepub
